# Biomarkers in clinical epidemiology studies

**DOI:** 10.1093/ckj/sfae130

**Published:** 2024-04-25

**Authors:** Carmine Zoccali, Giovanni Tripepi, Vianda Stel, Edouard L Fu, Francesca Mallamaci, Friedo Dekker, Kitty J Jager

**Affiliations:** Renal Research Institute, New York, USA; Institute of Molecular Biology and Genetics (Biogem), Ariano Irpino, Italy; Associazione Ipertensione Nefrologia Trapianto Renale (IPNET), c/o Nefrologia, Grande Ospedale Metropolitano, Reggio Calabria, Italy; CNR-IFC, Institute of Clinical Physiology, Research Unit of Clinical Epidemio logy; ERA Registry, Amsterdam UMC location and the University of Amsterdam, Department of Medical Informatics, Amsterdam, The Netherlands; Amsterdam Public Health, Quality of Care, Amsterdam, The Netherlands; Department of Clinical Epidemiology, Leiden University Medical Center, Leiden, The Netherlands; CNR-IFC, Institute of Clinical Physiology, Research Unit of Clinical Epidemio logy; Department of Clinical Epidemiology, Leiden University Medical Center, Leiden, The Netherlands; Department of Clinical Epidemiology, Leiden University Medical Center, Leiden, The Netherlands; Department of Clinical Epidemiology, Leiden University Medical Center, Leiden, The Netherlands; Nephrology, Dialysis and Transplantation Unit, Azienda Ospedaliera “Bianchi-Melacrino-Morelli” Grande Ospedale Metropolitano of Reggio Calabria, Italy

**Keywords:** biomarkers, cardiovascular, CKD, epidemiology, inflammation

## Abstract

This paper discusses the use of biomarkers in clinical practice and biomedical research. Biomarkers are measurable characteristics that can be used to indicate the presence or absence of a disease or to track the progression of a disease. They can also be used to predict how a patient will respond to a particular treatment. Biomarkers have enriched clinical practice and disease prognosis by providing measurable characteristics that indicate biological processes. They offer valuable insights into disease susceptibility, progression, and treatment response, aiding drug development and personalized medicine. However, developing and implementing biomarkers come with challenges that must be addressed. Rigorous testing, standardization of assays, and consideration of ethical factors are crucial in ensuring the reliability and validity of biomarkers.

Reliability is vital in biomarker research. It ensures accurate measurements by preventing biases and facilitating robust correlations with outcomes. Conversely, validation examines which and how many biomarkers correspond to theoretical constructs and external criteria, establishing their predictive value. Multiple biomarkers are sometimes necessary to represent the complex relationship between exposure and disease outcomes accurately.

Susceptibility factors are pivotal in disease states' complex interaction among genetic and environmental factors. Gaining a comprehensive understanding of these factors is essential for effectively interpreting biomarker data and maximizing their clinical usefulness. Using well-validated biomarkers can improve diagnoses, more effective treatment evaluations, and enhanced disease prediction. This, in turn, will contribute to better patient outcomes and drive progress in medicine.

According to the Food and Drugs Administration [1], biomarkers are defined characteristics measured as indicators of normal biological processes, pathogenic processes, or responses to an exposure or intervention, including therapeutic interventions. Molecular, histologic, radiographic, or physiologic characteristics are types of biomarker. This broad definition encompasses physical measurements such as blood pressure or left ventricular mass, and gene markers and metabolites measurable in the circulation or accessible body fluids and tissues.

The development and implementation of valid biomarkers in clinical practice have significantly affected the diagnosis, prognosis, and treatment of various diseases, including cancer, cardiovascular disease, and neurological disorders. Biomarkers also play a critical role in drug development because they can be used to identify patients who are most likely to respond to a specific therapy or to monitor the efficacy and safety of a treatment. This review mainly focuses on serum or plasma biomarkers and will discuss the different types of biomarker, their role in disease diagnosis and treatment, and the challenges associated with their development and implementation in clinical practice in the context of CKD.

Biomarkers can be classified into several categories on the basis of their sources, such as genomic, proteomic, or metabolomic biomarkers, or their clinical utility, such as diagnostic, prognostic, or predictive biomarkers. Genomic biomarkers, such as mutations or gene expression profiles, can provide valuable information on disease susceptibility, progression, and response to treatment. Proteomic and metabolomic biomarkers, such as protein or metabolite levels, can reflect changes in biological pathways and metabolic processes associated with disease. Diagnostic biomarkers can identify the presence or absence of a disease, while prognostic biomarkers can provide information on disease progression and outcome. In theory, predictive biomarkers can be used to select patients who are most likely to benefit from a specific therapy, providing the basis for ‘precision medicine’. For example, in the 2021 KDIGO guidelines for managing glomerular diseases, the level of PLA2-R antibodies is considered useful for diagnosing and guiding therapy in primary membranous nephropathy [2].

The development and use of biomarkers demand undertaking. Biomarker validation requires rigorous testing in large, diverse patient populations and the development of standardized assays and analytical methods. Furthermore, biomarker implementation requires careful consideration of ethical, legal, and social issues, such as patient privacy and confidentiality, informed consent, and access to testing.

Twenty-five years ago, the Committee on Biological Markers of the National Research Council/National Academy of Sciences broadly categorized biomarkers into markers of exposure, effect, and susceptibility [3]. This concept set the stage for discussing biomarkers' potential and limitations in epidemiological research [4, 5]. In this review, we adopted the conceptual framework by Schulte to discuss biomarkers in epidemiological research [5]. Biologic markers are helpful in etiologic and mechanistic research, secondary disease prevention, risk assessment, and therapeutic effectiveness. We will produce examples related to chronic kidney disease and hypertension and, more sparsely, to diabetes and metabolic diseases.

## THE CONCEPTUAL FRAMEWORK UNDERLYING THE USE OF BIOMARKERS IN CLINICAL AND EPIDEMIOLOGICAL RESEARCH

The conceptual framework for research strategies for using biological markers in epidemiologic research revolves around studies to validate and characterize relationships between the various classes of biomarkers. Preliminarily, it is worth emphasizing that biomarkers per se cannot be used to assess causality, an issue that can only be solved with a well-targeted intervention.

**Figure 1:**
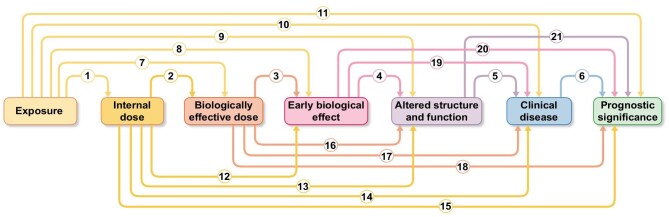
Possibilities of application of biomarkers in research across the exposure–prognosis series. Internal dose (the quantity of the toxic exposure, be it an endogenous factor such as hyperglycaemia, or external factors such as air pollutants) may predict the clinical disease and prognosis. Each element of the exposure–prognosis pathway can be analysed in the relationship to the other elements. In all, there are 21 possibilities.

As shown in Fig. 1, the exposure–disease continuum is conceptualized as a temporal sequence of seven generic components. These components include exposure; internal dose, i.e. the quantity of the toxic exposure, be it an endogenous factor such hyperglycaemia, or external factors such air pollutants found in the blood or another biological medium; biologically effective dose, i.e. the amount of the toxic factor actually affecting sensitive targets at subcellular, cellular, and tissue levels; early biological effect, i.e. an early event associated, and often predictive of an alteration of the health status (for example, microalbuminuria in hyperglycaemia); and altered structure/function, i.e. a recognizable prodromal phase of the disease. Finally, markers of disease and prognostic markers refer to markers that establish the diagnosis and predict the disease in question (Fig. 1). Figure 1 illustrates the 21 possible nominal relationships that can be evaluated along the continuum between exposure and disease.

The linear causal sequence is an implied framework in most exposure–disease relationships. Still, the continuum paradigm is only meant to elucidate a single pathway among many to a disease from a given exposure. Multiple pathways leading to a given disease should be considered, and the contribution of one due to a specific exposure needs to be evaluated considering contributions from other pathways resulting from other exposures. For example, in CKD, hypertension is a noxious exposure that operates together with other pathways in causing renal damage, such as inflammation or age-related processes (senescence-related factors).

For research planning purposes, one way to conceptualize the possibilities is to consider markers of exposure [exposure (initial), internal dose, or biological effective dose] and markers of effect (altered structure and function, clinical disease, and prognosis) as categories. The goal would be to initiate research that examines the possible link between a marker of exposure and a marker of effect and to determine the entire continuum for a certain exposure.

The heuristic linear sequential model should not preclude efforts to explore more complex relationships between markers. Multiple markers may be more efficacious than a single marker for characterizing a continuum component. The importance of the 21 possible relationships between any two components in the exposure–disease continuum (including markers of prognostic significance) depicted in Fig. 1 will vary depending on the priorities and objectives of investigators and funding institutions. Table 1 shows how some objectives can be met by studying the associated relationships. These are not the only relationships that can be studied to meet those objectives, but they represent possible initial approaches.

**Table 1:** Examples of the use of biomarkers for understanding the effect of toxic environmental exposures.

Exposure	Internal dose	Biologically effective dose	Early biologic effect	Alterations in structure and/or function	Disease	Prognosis
Lead	Blood lead levels	Lead levels in the bone marrow cells	Inhibition of the D-aminolaevulinic acid dehydratase	Accumulation of Zn-protoporphyrin	Anaemia	Rate of lead decrease after cessation of the exposure
Fatty foods	Serum cholesterol	LDL/HDL	Blood diamicrons	Serum enzymes of myocardial necrosis	Atherosclerosis	Serum enzymes of myocardial necrosis
Ethylene dioxide	Haemoglobin adducts	DNA adducts	Hypoxanthine guanine phosphoribosyl transferase mutation	Sister chromatid exchange	Leukaemia	?
Dioxin	$2,3,7,8$ -tetrachloro- dibenzo-p-dioxin	Urinary porphyrins	Hyperkeratinization of sebaceous glands	?	Chloracne	?

Biomarkers in the exposure–disease continuum can be applied like ‘exposure’ or ‘disease’ in experimental research. Still, this approach may be problematic if the same biomarkers do not reflect ‘critical effects’ or adequately represent the considered component. Determining the critical effect among various effects requires independent basic studies and confirmation in clinical and epidemiologic research. Once critical effects are established, they must be related to estimates of the amount (dose) of preceding and succeeding components in the continuum. Reliability and validation are essential aspects when working with biological markers.

## RELIABILITY AND VALIDATION OF BIOMARKERS

A biomarker's reliability refers to controlling measurement errors, which, if left uncontrolled, can lead to decreased sensitivity and other untoward consequences. Conducting a pilot reliability study before major research and replicating measurement procedures can improve reliability.

Validation of biological markers can be viewed in terms of construct, content, and criterion validity [6]. Construct validity is the ability of a marker to correspond to theoretical constructs under study, i.e. how well the biomarker correlates with relevant characteristics of the phenomenon being investigated, for example, how well a biochemical marker (e.g. creatinine) correlates with actual kidney damage. In other words, the biomarker will be positive or negative (high or normal creatinine) for some relevant characteristics of the phenomena, such as another test (degree of inflammation or kidney fibrosis on histology), and the results of the study will be the correlation or agreement between the two measures. It should be emphasized that, per se, the construct validity does not guarantee that the biomarker–phenomenon relationship, whatever its strength, is not proof of causality. In this respect, the randomized clinical trial remains the decisive proof for assessing the causal involvement of biomarkers in the aetiology of diseases. Content validity broadly pertains to biological phenomena the biomarker is expected to reflect. This is a weak criterion because it is based on professional judgement or consensus of the field. This criterion is usually not considered in validating laboratory techniques, and its application is restricted to social sciences. For example, content validity is important in studies examining patient-reported outcomes. Criterion validity is how the measurement correlates with an external criterion of the phenomenon under study, that is, whether the biomarker predicts an aspect of the phenomenon under study. Sensitivity, specificity, and predictive value are the typical instruments to assess criterion validity. Two types of criterion validity are distinguished. Concurrent validity is when the biomarker and the criterion are applied at the same time. For example, exposure to an air pollutant can be validated by measuring the same pollutant in the air and the breath of patients. Predictive validity is the biomarker's ability to predict the disease's occurrence; for example, a genetic biomarker such as the apoprotein L1 gene variant in African-Americans that predicts a high risk for CKD.

Validation of the relationship between various components of the continuum from exposure to a disease involves four levels of effort: determination of an association between a marker and preceding exposure or subsequent effect; location, shape, and slope of the exposure–marker or marker–effect relationship; threshold of ‘no observed effect’ level; and positive predictive value of the marker for exposure or disease [7]. The ultimate criterion of a marker is whether it has a strong positive predictive value. A successful biological marker of effect should identify those most likely to become diseased among all exposed individuals.

## MULTIPLE MARKERS

In 2005, a group of tumour biomarker research experts introduced the Reporting Recommendations for Tumour Marker Prognostic Studies (REMARK) criteria. These guidelines and the Biospecimen Reporting for Improved Study Quality criteria offer a structured approach for transparently reporting study methods and analyses [8]. Altman and Lyman have categorized research about the evaluation of biomarkers for prediction into four phases, starting from exploratory biomarker investigations with the purpose of selecting a few biomarkers from many, over exploratory associational studies with few biomarkers, confirmative associational studies to prediction model development [9]. Several markers are sometimes needed to represent a component in the exposure–outcome relationship accurately. For example, to robustly investigate the relationship between inflammation and CKD progression, Amdur *et al*. measured the multivariable association of plasma levels of IL-1, IL-1 receptor antagonist, IL-6, TNF-α, TGF-β, high-sensitivity C-reactive protein, fibrinogen, and serum albumin with the progression of CKD in 3430 Chronic Renal Insufficiency Cohort study participants [10]. These biomarkers reflect different inflammatory pathways. While IL-1β is an inflammasome-dependent cytokine, IL-6 and IL-α are inflammasome-independent. TNF-alpha is mainly produced by activated macrophages, T lymphocytes, and natural killer cells. C-Reactive Protein, fibrinogen, and albumin are acute-phase reactants produced in the liver during acute and chronic inflammatory states. Testing biomarkers of these inflammatory pathways provides more comprehensive testing of the inflammation vs CKD progression relationship. IL-1 and IL-6 predicted the study outcome, indicating that both inflammasome-dependent and -independent pathways contribute to progressive renal damage in CKD. The selection of inflammatory biomarkers in the CRIC study had solid biological underpinnings. However, two significant issues arise when using multiple markers: (i) how to select the best markers from multiple candidates and (ii) how to combine them into a helpful index. Applying discrimination rules, such as linear discrimination, logistic discrimination, quadratic discrimination, and recursive partitioning, is a rational solution [11]. Also, correlated markers can be used and simultaneously tested in multivariable models.

## SUSCEPTIBILITY

**Figure 2:**
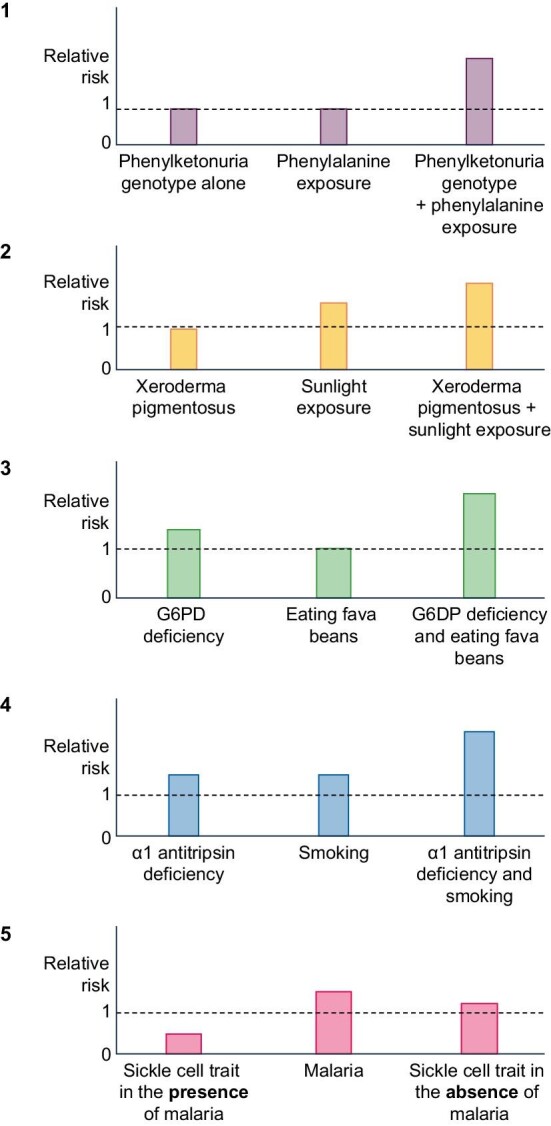
The risk of disease for genetic markers depends on the intensity of exposure to environmental factors, the strength of genotype–environment interactions, and the nature of the environmental effect in relation to the genotype. Reference risk is the risk in the undiseased population (the broken line). Six patterns of genetic and environmental interactions can influence this relationship. The first pattern involves the combined effect of genotype and exposure causing excess risk (e.g. phenylalanine and phenylketonuria leading to mental retardation). The second pattern is when an innocuous genotype is affected by an environmental trigger (e.g. xeroderma pigmentosa and sunlight-induced skin cancer). The third pattern shows a genotype associated with risk (G6PD deficiency), while the environmental exposure alone, i.e. eating fava beans carries no excess risk. The fourth pattern involves both genotype and environment contributing to disease risk (e.g. α-1 antitrypsin deficiency and smoking in pulmonary emphysema). The fifth pattern shows how the genotype's effect changes with the presence or absence of an environmental factor (e.g. sickle cell trait being protective against malaria but harmful in its absence).

Susceptibility is another crucial aspect to consider when evaluating the association between two components in the continuum. Susceptibility depends on various genetic or acquired host factors. The relative risk of disease for genetic markers is a function of the intensity of exposure to the environmental factor, the strength of interaction between the genotype and the environmental factor, and the nature of the environmental effect in relation to the genotype (specific vs unspecific). Six patterns of genetic and environmental interactions can influence the relationship between a genetic marker and a disease [12] (Fig. 2). In the first pattern, the genotype and exposure alone do not cause excess risk for adverse health outcomes. For example, phenylalanine in the diet and its interaction with the phenylketonuria genotype in causing mental retardation. In this example, neither exposure to phenylalanine nor genotype alone produces excess disease risk, while their combination does. The second interaction pattern is between an innocuous genotype without specific exposure and an environmental exposure effect in individuals without the genotype. An example of this interaction is that between xeroderma pigmentosa and exposure to sunlight and the production of skin cancer. In this case, the genotype requires an environmental trigger (UV light), while sunlight is a risk factor for skin cancer regardless of the presence of xeroderma pigmentosa. In the third pattern, the genotype per se is associated with excess risk, whereas the exposure alone is not. For example, eating fava beans alone does not produce haemolytic anaemia, whereas G6PD deficiency does if exposed to certain antimalarial drugs. In the fourth pattern, the genotype and the environment alone are associated with the disease. This is the case of α-1 antitrypsin deficiency and cigarette smoking in pulmonary emphysema. Individuals with α-1 antitrypsin deficiency have a very high risk of chronic obstructive pulmonary disease independently of environmental risk factors (e.g. smoking). Smokers have a high risk of chronic obstructive pulmonary disease even without α1-anti-trypsin deficiency. The fifth pattern of interaction occur when the genotype's effect is reversed depending on the environmental factor's presence (fifth pattern) For example, the sickle cell trait may be advantageous in the presence of malaria but disadvantageous in the absence of this disease.

## EPIDEMIOLOGICAL DESIGNS

Observational studies are the second step in the evidence ladder for investigating the associations between risk factors and diseases [13]. These studies, especially cohort studies based on repeated measurements of the predictor variable, are valuable for assessing causality and often the results of these studies are confirmed by the results of clinical trials looking at the same problem [14]. However, various problems of observational epidemiology strongly limit its ability to establish causal effects. These include: (i) reverse causation, i.e. observational contexts where the outcome affects the exposure; (ii) confounding, when shared causes among the risk factor and the outcome coexist; (iii) selection bias, when cohort participants are selected in a manner that prejudices the correct estimate of the effect; and (iv) imprecisions in the measurement of the exposure or confounding factors or outcome. In cohort studies, various instruments can be adopted to explore the link between risk factors and diseases [3]. Designs different from cohort studies, namely cross-sectional and case-control studies, cannot assess causal hypotheses [15]. However, these designs are certainly valid in some contexts, as was the case for Factor 5 Leiden [16]. Longitudinal studies are the preferred observational approach for exploring causality in the observational scenario. Indeed, these studies are centred on the temporal sequence, from the exposure component to the prognostic component in Fig. 1. Longitudinal studies are time- and resource-intensive. In a longitudinal study, biomarker components in the continuum can be independent variables for any component biomarker (dependent variable) to its right in the continuum (Fig. 1). It should be noted that, however valid and robust, longitudinal studies are inherently insufficient for establishing causality. Thus, a biomarker that demonstrates good performance in a longitudinal study should eventually be tested in a randomized clinical before being recommended for large-scale application in clinical practice.

In conclusion, biomarkers are invaluable tools in clinical practice and biomedical research. They provide insights into disease processes, aid in diagnosis, prognosis, and treatment response, and facilitate drug development and personalized medicine. Biomarker studies may also be useful for prediction studies and screening. However, the reliability and validation of biomarkers are essential considerations. Ensuring accurate measurements and assessing the biomarker's correspondence to theoretical constructs and external criteria are crucial for their effectiveness and clinical utility [8, 9]. Challenges such as diverse patient populations, standardized assays, and ethical issues must be addressed in biomarker development and implementation. By improving our understanding of biomarker reliability and validation, we can harness their full potential to enhance disease management, optimize treatment outcomes, and drive advancements in biomedical research to benefit patients.

## Data Availability

The data underlying this article are available in the article itself.
