# Correction to: Biomarkers in clinical epidemiology studies

**DOI:** 10.1093/ckj/sfae293

**Published:** 2024-09-28

This is a correction to: Carmine Zoccali, Giovanni Tripepi, Vianda Stel, Edouard L Fu, Francesca Mallamaci, Friedo Dekker, Kitty J Jager, Biomarkers in clinical epidemiology studies, *Clinical Kidney Journal*, Volume 17, Issue 6, June 2024, sfae130, https://doi.org/10.1093/ckj/sfae130

In the originally published version of this manuscript, there was a typographical error in the name of the fourth-listed author, Edouard L. Fu.

This has been corrected.

